# The Marine Hospital Service

**Published:** 1879-01

**Authors:** 


### ARTICLE XIII.

### THE MARINE HOSPITAL SERVICE.

Those of our readers who have observed the changes that have been lately wrought in the U. S. Marine Hospital service, must have been greatly encouraged by the reflections they suggest. Time was when its affairs were in a state of utter confusion and chaos. They were administered in the interest of politicians, by officers who were themselves the creatures of scheming congressmen, and in most instances, instead of being selected on account of their fitness to perform the duties to which they were appointed, the reason for their appointment was that they or their friends had been, or might become, useful in some political campaign.

As a consequence of the want of system and organization and the incompetence of official incumbents, the service was a great expense to the United States Treasury. Hundreds of thousands of dollars were annually expended without the poor sailors receiving a moiety of the benefit that is now realized from the same expenditure. Great praise is due to ex-Senator Logan, in this connection, for the effective work he performed in congress. It was through his influence mainly that the office of Surgeon-General of the Marine Hospital Service was created, and the present efficient occupant of that office selected. No one can doubt the wisdom of the course pursued by Senator Logan, either in reference to the office he aided in creating or in the selection of Dr. John M. Woodworth to worthily fill it.

The marine hospital service, under Dr. Woodworth, has been literally reorganized. Its affairs are now administered honestly, wisely and economically. It is no longer an expense to the government, as it once was, but its income now covers the cost incurred by the excellent and efficient attention given to sick and disabled sailors.

It is only necessary to look at some of the measures the chief has inaugurated to see how all these good results have been accomplished. He has wrested the management of the service from politicians, and taken from them, all the power they formerly possessed in making appointments to its offices ; and this was no easy task as we happen to know from a somewhat intimate acquaintance with the matter. Dr. Woodworth passed through many an ordeal in his efforts in this direction, and more than once his friends had reason to fear that he would loose his official head. This, however, was a necessary step preliminary to success in obtaining a sound organization. The next thing to do was to secure intelligent and trustworthy subordinates. In place of a hap-hazard method, each applicant for a place in the service is now subjected to a rigid examination by a board of surgeons convened annually at Washington city. The examination extends over five days and consists in a thorough test of the professional qualification of all who aspire to become officers. This extends to their preliminary education and moral character, and also to clinical examinations at the bedside of the patient. Those passing the highest grade are appointed assistant surgeons.

All vacancies in the rank of surgeons are filled by promotion from the grade of assistant surgeons, on the grounds of merit and fitness alone.

These are some of the most important measures adopted by the Surgeon General to perfect the Marine Hospital Service ; others of minor consideration we do not consider it necessary to mention. In addition to the faithful and efficient discharge of these onerous duties connected with his office, and which are to a great extent self-imposed, Dr. Woodworth has found time to do a great deal for the profession at large. The corps of the Marine Hospital Service was the first to adopt the nomenclature promulgated by the Royal College of Physicians, London, which is now regarded by the profession generally as the best in use.

The officers of this branch of public service are now educating the profession of this country in the use of the metric system for dispensing medicines, so well adapted in every way to this purpose.

To the Supervising Surgeon-General also is due the credit of urging upon Congress the quarantine act. He is now issuing and distributing bulletins of epidemic disease throughout the world, and while much of the space in them is occupied by subjects connected with the advance, prevalence and decline of yellow fever in this country, still telegraphic intelligence in reference to that disease and to cholera, etc., from the most distant parts of Europe and the East occupies a place in the reports. It requires no spirit of prophecy to foretell the great advantage which must result from this comprehensive system of gathering information of the rise, progress and approach of future epidemics. Our quarantine authorities will not only know of the approach of an epidemic disease, but will be informed, by name and description, of the departure from an infected district or port of every vessel that may be destined for this country.

If we mistake not, the idea, inception and execution of the plan of forming a yellow-fever commission to collect all the facts in reference to the prevalence of that awful scourge of the South, originated also with the Marine hospital management. The good that will flow from the creation of this commission, and their report to the Public Health Association, recently held in Richmond, is not all. These and other circumstances that are now of importance to the profession, strongly suggest the benefits that would arise from a national bureau of public health. In fact, the foundations are laid for it in such a way that sooner or later its establishment will be secured. We deem it but just to lay before our readers this rapid and imperfect sketch of the work accomplished by our fellow townsman, Dr. John M. Woodworth. He has made an indelible impression upon the sanitary record of this country; while the history of the United States Marine Hospital Service will point to his exertions in its behalf in all time to come. His is a deservedly prominent position, and one of which the nation should be proud.

